# Supplementary material for: Mechanisms governing the pioneering and redistribution capabilities of the non-classical pioneer PU.1
Source: Nat Commun. 2020 Jan 21;11:402. doi: 10.1038/s41467-019-13960-2 (PMC6972792; doi:10.1038/s41467-019-13960-2)
Supplement: Supplementary file 7 — Source data [file 41467_2019_13960_MOESM7_ESM.zip › Source_Data/FigureS2/FigureS2B_MotifScanOutput/KmeansCluster2/homerResults/motif24.similar.html]

motif24

## Information for motif24

C
G
T
A
A
C
T
G
C
G
A
T
A
C
G
T
A
G
T
C
A
C
G
T
A
G
T
C
C
T
G
A
A
G
C
T
C
T
A
G
C
G
T
A
A
C
T
G
C
G
T
A
  
Reverse Opposite:  

A
C
G
T
G
T
A
C
A
C
G
T
A
G
T
C
C
T
G
A
A
G
C
T
A
C
T
G
C
G
T
A
A
C
T
G
C
G
T
A
C
G
T
A
A
G
T
C
C
G
A
T
  

|  |  |
| --- | --- |
| p-value: | 1e-50 |
| log p-value: | -1.159e+02 |
| Information Content per bp: | 1.923 |
| Number of Target Sequences with motif | 150.0 |
| Percentage of Target Sequences with motif | 2.99% |
| Number of Background Sequences with motif | 209.3 |
| Percentage of Background Sequences with motif | 0.51% |
| Average Position of motif in Targets | 102.7 +/- 42.3bp |
| Average Position of motif in Background | 95.8 +/- 69.9bp |
| Strand Bias (log2 ratio + to - strand density) | -0.0 |
| Multiplicity (# of sites on avg that occur together) | 1.22 |
| Motif File: | file (matrix) reverse opposite |

### Similar de novo motifs found

|  |  |  |  |  |  |  |  |
| --- | --- | --- | --- | --- | --- | --- | --- |
| Rank | Match Score | Redundant Motif | P-value | log P-value | % of Targets | % of Background | Motif file |
| 1 | 0.941 | A T G C C G T A C T A G C G A T A C G T T G A C G A C T A G T C T C G A A C G T T C A G C G T A C A T G T C G A | 1e-46 | -106.861320 | 3.84% | 0.96% | motif file (matrix) |
| 2 | 0.923 | C T A G G C A T A C G T G T A C G C A T A G T C T C G A A G C T T C A G C G T A A C T G C T G A | 1e-44 | -101.625092 | 4.28% | 1.22% | motif file (matrix) |
| 3 | 0.771 | G A C T A T G C C T A G G A C T T A C G C T G A A C T G C T G A C T G A G A T C | 1e-31 | -72.457892 | 1.79% | 0.30% | motif file (matrix) |
